# Hashimoto's thyroiditis presenting as a single toxic adenoma (A case report)

**DOI:** 10.22088/cjim.11.4.450

**Published:** 2020

**Authors:** Mehrnoush Sohrab, Zahra Kashi, Adele Bahar

**Affiliations:** 1 Diabetes research center, Mazandaran University of Medical Sciences, Sari, Iran

**Keywords:** Hypothyroidism, Hashimoto's thyroiditis, Hot nodule

## Abstract

**Background:**

Hashimoto's thyroiditis can be present with a localized palpable nodule though presentation as a hyperfunction "nodule" is extremely rare. The first case of Hashimoto's thyroiditis and hot nodule was reported in 1971 by Warner.

**Case Presentation:**

We reported a 26-year-old hypothyroid woman in Hashimoto's thyroiditis background with a hyperactive thyroid nodule in both 99mTc and I_131_scintigraphy. The nodule disappeared after eight months of sufficient thyroid hormone replacement therapy.

**Conclusion:**

Toxic adenoma in hypothyroid patients can be resolved after levothyroxine (L-T4) replacement therapy.

Hashimoto's thyroiditis (HT) is the most common cause of hypothyroidism in iodine-sufficient areas. Hypothyroidism is seen in up to 10 percent of the population, and its prevalence increases with age (1). Although occasional patients with Hashimoto's thyroiditis present with a localized palpable "nodule," these are usually hypofunctioning or "cold" areas on the scan. Presentation as a functioning "nodule" is extremely unusual. The first case of Hashimoto's thyroiditis presents as a solitary functioning thyroid "nodule" was reported by Warner et al. in 1971 (2). This rare condition has been previously reported in few cases too. The scintigraphic findings of HT are highly variable and can mimic several thyroid disorders including diffuse hyperplasia, nodular goiter, cold nodules, and rarely hot nodules (3). We present a 26-year-old woman with severe hypothyroidism since adolescence associated to single hot Tc-99 pertechnetate thyroid nodule. 

## Case presentation

A 26-year-old woman with family history of hypothyroidism on her mother side was referred to our endocrine clinic because of clinical hypothyroidism and a thyroid hot nodule (table 1). After hypothyroidism diagnosis, the thyroid hormone replacement (500 micg/week) has been started and a thyroid sonography requested due to the feeling of lump in the neck by the patient. The ultrasonography findings were an enlarged thyroid gland with diffusely hypo echogenic pattern and heterogeneous echo texture. A 13*6 mm solid isoechoic nodule with regular and sharp margin has been detected in the thyroid isthmus without significant lymphadenopathy. Her first physician had requested a sonography guided fine needle aspiration cytology (FNAC) from the thyroid nodule. The cytology showed a hyper cellular smear containing monolayer sheets, nests, micro follicles and mild nuclear follicular cells pleomorphism in addition to pigment laden macrophages and multinucleated giant cell in hemorrhagic and colloid background.

**Table 1:** Laboratory test at presentation and after 6 months follow-up

**Lab test**	**The first visit**	**After 6 months follow-up**
TSH (0.3-5.6mU/L)	20	2.2
T4 (4.6-12 Ug/dl)	2	8.1
T3		1.1
Anti Tpo (<40 Iu/ml)	410	

**Figure 1:**
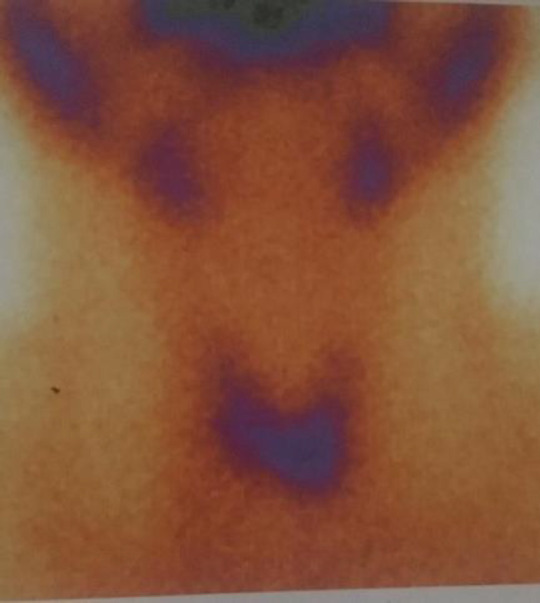
Thyroid scan Technetium 99 scintigraphy scan

The final cytological diagnosis was “suggestive of thyroid neoplasm”. The patient was referred to our clinic because of hot area in the thyroid gland isthmus (compatible with thyroid nodule) according to 99mTc pertechnetate thyroid scintigraphy report. Residual thyroid tissue had low uptake (figure 1). 

**Figure 2:**
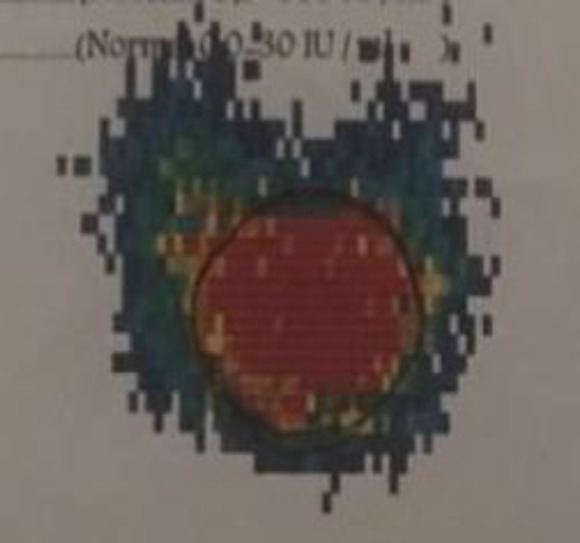
Thyroid scan

**Figure 3:**
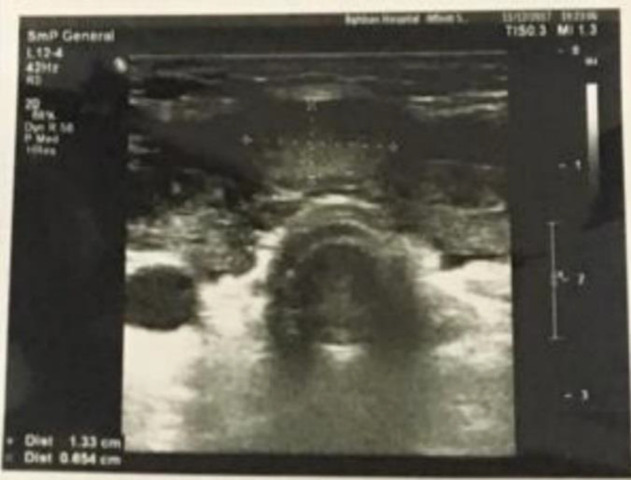
The first thyroid sonography

We requested an I131 thyroid scintigraphy to rule out the false hot nodule report by 99mTc scintigraphy, which confirmed the hot condition of nodule (figure 2). So we decreased the thyroid hormone replacement dosage for definitive assurance of the hypothyroidism diagnosis. Three months after reduced drug doses, the TSH level increased to 25 IU/L. Color flow Doppler ultrasonography performed by a thyroid ultrasound specialist revealed a solid hypoechoic nodule of approximately 21*19*9 mm in diameters (compared to first sonography which the nodule was isoechoic and lower in size) with well demarcated margin. Increased blood flow was reported in thyroid nodule periphery in addition to thyroid parenchyma (figure 3).

**Figure 4:**
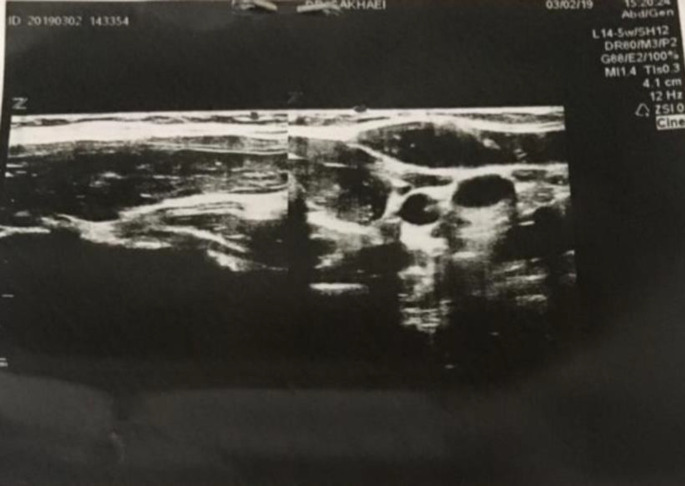
The last thyroid sonography

L-T4 substitution therapy was started again with full dose (700 micg/week) and fine needle aspiration (FNAC) was repeated because of increase in nodule size. Cytology report revealed an adenomatous nodule in background of lymphocytic thyroiditis. The thyroid function tests were acceptable in follow-up .The nodule size diminished to 11*5 mm at the four months full dose levothyroxine replacement therapy and disappeared after 8 months follow-up according to sonography report (figure 4).

## Discussion

Hashimoto thyroiditis is the most common cause of primary hypothyroidism in rich iodine area. The diagnosis of Hashimoto thyroiditis can be confirmed when a patient with hypothyroidism had firm consistency thyroid on palpation and high level of thyroid peroxidase antibody (4).

We reported a young hypothyroid female due to Hashimoto thyroiditis and a thyroid hot nodule in scintigraphy which disappeared with sufficient thyroid hormone therapy. Although there are numerous reports about thyroid nodule associated with Hashimoto's thyroiditis, but the association of hypothyroidism in Hashimoto thyroiditis background with thyroid hot nodule is rare. Scintigraphic findings in Hashimoto thyroiditis are very different such as multinodular goiter, diffuse hyperplasia and some of them mimic solitary nodule but the toxic adenoma is rare condition (4). To the best of our knowledge, eighteen Hashimoto thyroiditis cases with thyroid toxic adenoma were reported through literature. Most patients were middle-aged women. In our case report, the Tc99m and I131 thyroid scintigraphy were concordant and reported a hyperactive thyroid isthmus nodule. This was the same as in the reports of Boughattas (3 case), Mousavi (1 case) and Zengi (1 case) (5-7). In the remaining reported cases (13 case) the Tc99m and I scintigraphy scan were not concordant (7). An increase in intrathyroidal vascularity and blood velocity are seen in Graves hyperthyroidism (8, 9) Autonomous functioning thyroid nodules can be seen as a solid iso-hypoechoic nodule with internal hyper vascularization (type III pattern) on thyroid color Doppler sonography (8, 10). This finding has been reported in the Hashimoto thyroiditis, too (9). This phenomenon is attributed to increased TSH level in these patients [9] and it is suggested that the chronic thyrotropin stimulation may be cause of hot nodule formation in Hashimoto thyroiditis (11). Thyroid ultrasonography in our patient found a well-defined, solid hypo echo nodule in the isthmus.In Color Doppler sonography, increased vascularity in periphery of nodule and thyroid parenchyma was seen. It was the same as Zengi and Boughattas’ for hyper vascularity in nodule but in our case,thyroid parenchyma was hypervascular against the Zengi and Boughattas’ report in which the remaining thyroid tissue was hypo echogenic and hypo vascular (5, 7).

The first US guided fine needle aspiration cytology (FNAC) report was suggestive of thyroid neoplasm whereas the second FNAC after 6 months reported an adenomatous nodule in a background of lymphocytic thyroiditis. Only available cytology report by Mousavi et al. in three cases showed normal follicular cells with lymphocytic infiltration in two of them (6) and in Zengi’s reported case, the cytology of nodule revealed normal follicular cells and colloid (7). 

In some reports after six months follow-up, the nodule size decreased between 40-60 % on adequate thyroid hormone replacement therapy (6, 7, 11) which was the same as our hypothyroid patient. In the last thyroid sonograghy of ours, the nodule disappeared after hypothyroidism correction. It may be due to the hyper stimulation effect of elevated TSH according to Zantour hypothesis (11).

In conclusion thyroid adequate replacement therapy has been advised for hypothyroid patients in Hashimoto thyroiditis background and toxic adenoma. 
